# Scaling Up Safer Birth Bundle Through Quality Improvement in Nepal (SUSTAIN)—a stepped wedge cluster randomized controlled trial in public hospitals

**DOI:** 10.1186/s13012-019-0917-z

**Published:** 2019-06-19

**Authors:** Rejina Gurung, Anjani Kumar Jha, Susheel Pyakurel, Abhishek Gurung, Helena Litorp, Johan Wrammert, Bijay Kumar Jha, Prajwal Paudel, Syed Moshfiqur Rahman, Honey Malla, Srijana Sharma, Manish Gautam, Jorgen Erland Linde, Md Moinuddin, Uwe Ewald, Mats Målqvist, Anna Axelin, Ashish KC

**Affiliations:** 1 Golden Community, Jwagal, Lalitpur, Nepal; 2 Ministry of Health and Population, Government of Nepal, Kathmandu, Nepal; 3 Nepal Health Research Council, Ramshah Path, Kathmandu, Nepal; 4 Department of Women’s and Children’s Health, Uppsala University, Dag Hammarskjölds väg 14B, Uppsala, Sweden; 5 Anweshan, Lalitpur, Nepal; 6 Department of Paediatrics, Stavanger University Hospital, Våland burrough, Stavanger, Norway; 7 Maternal and Child Health Division, ICDDR,B, Dhaka, Bangladesh; 8 University of Turku, Turku, Finland; 9 Society of Public Health Physicians Nepal, Lalitpur, Nepal

**Keywords:** Quality improvement interventions, Basic neonatal resuscitation, Fetal heart rate monitoring, Stepped wedge cluster randomized control trial, Nepal

## Abstract

**Background:**

Each year, 2.2 million intrapartum-related deaths (intrapartum stillbirths and first day neonatal deaths) occur worldwide with 99% of them taking place in low- and middle-income countries. Despite the accelerated increase in the proportion of deliveries taking place in health facilities in these settings, the stillborn and neonatal mortality rates have not reduced proportionately. Poor quality of care in health facilities is attributed to two-thirds of these deaths. Improving quality of care during the intrapartum period needs investments in evidence-based interventions. We aim to evaluate the quality improvement package—Scaling Up Safer Bundle Through Quality Improvement in Nepal (SUSTAIN)—on intrapartum care and intrapartum-related mortality in public hospitals of Nepal.

**Methods:**

We will conduct a stepped wedge cluster randomized controlled trial in eight public hospitals with each having least 3000 deliveries a year. Each hospital will represent a cluster with an intervention transition period of 2 months in each. With a level of significance of 95%, the statistical power of 90% and an intra-cluster correlation of 0.00015, a study period of 19 months should detect at least a 15% change in intrapartum-related mortality. Quality improvement training, mentoring, systematic feedback, and a continuous improvement cycle will be instituted based on bottleneck analyses in each hospital. All concerned health workers will be trained on standard basic neonatal resuscitation and essential newborn care. Portable fetal heart monitors (Moyo®) and neonatal heart rate monitors (Neobeat®) will be introduced in the hospitals to identify fetal distress during labor and to improve neonatal resuscitation. Independent research teams will collect data in each hospital on intervention inputs, processes, and outcomes by reviewing records and carrying out observations and interviews. The dose-response effect will be evaluated through process evaluations.

**Discussion:**

With the global momentum to improve quality of intrapartum care, better understanding of QI package within a health facility context is important. The proposed package is based on experiences from a similar previous scale-up trial carried out in Nepal. The proposed evaluation will provide evidence on QI package and technology for implementation and scale up in similar settings.

**Trial registration number:**

ISRCTN16741720. Registered on 2 March 2019.

## Background

The accelerated reduction in maternal and child mortality during the Millennium Development Goal era (2000–2015) led to the realization that further reduction can only be achieved with improved quality of care in the intrapartum period [1, 2]. Every year, almost 1.2 million stillbirths and 250,000 maternal deaths occur during the intrapartum period, and a million newborns die in their first day of life [3–5]. The United Nations’ Every Woman and Every Child strategy 2016–2030 aims to reduce preventable maternal, neonatal, and child deaths by the end of the Sustainable Development period 2030 [6]. The strategy has resulted in a number of efforts to identify ways of reducing preventable deaths [7]. One of the key initiatives was led by the Lancet Global Health Commission for High Quality Health Systems in the SDG Era [8]. Through consultations and systematic reviews, the commission produced a framework for improved quality of care in health care settings [9, 10]. Its five foundations are (1) understanding populations’ health care needs; (2) strengthening structures and governance for improving quality of care at all levels of health systems [11]; (3) redesigning and optimizing the health workforce to provide a more conducive environment for health care provision; (4) introducing new tools, quality improvement interventions, and technologies for delivering health care; and (5) the adequacy or capacity of health facilities to deliver health care as per the demands of their client populations [8].

The PARiHS framework (Promoting Action on Research Implementation in Health Services) promotes the translation of evidence into practice using context and facilitation as interplay for improving quality of care [12, 13]. This is a useful framework as two-thirds of premature and preventable neonatal death are due to poor quality health care and *not to* lack of access to health care [14]. The large investments made in improving access to and the availability of maternal and newborn care since the start of the MDG period in 2001 [15] had a large impact on reducing the extent of the first and second delays of maternal and newborn care [16]. However, the third delay of inadequate quality of care remains a large challenge [17, 18].

In Nepal, several studies have found inadequate quality of care as a major barrier to reducing maternal and neonatal deaths, especially during the intrapartum period [19, 20]. The following approaches for quality improvement have been effective in low-income settings to overcome this barrier [21–23]:

- Understanding the context in which health facilities operate, and based on this improving leadership to bring about organizational improvements [24];
- Facilitating quality improvement through external and internal drivers such as mentoring and feedback from audits [25] for bringing improvements in clinical units [26].
- Introducing new standards and tools for improving efficiency to implement the standards.
- Setting up data platforms for instigating accountability for the provision of quality care [27].

The actual ‘dose-response’ of the above four approaches will depend upon the adequacy of QI package implementation and the context of implementation [28].

**Fig. 1:**
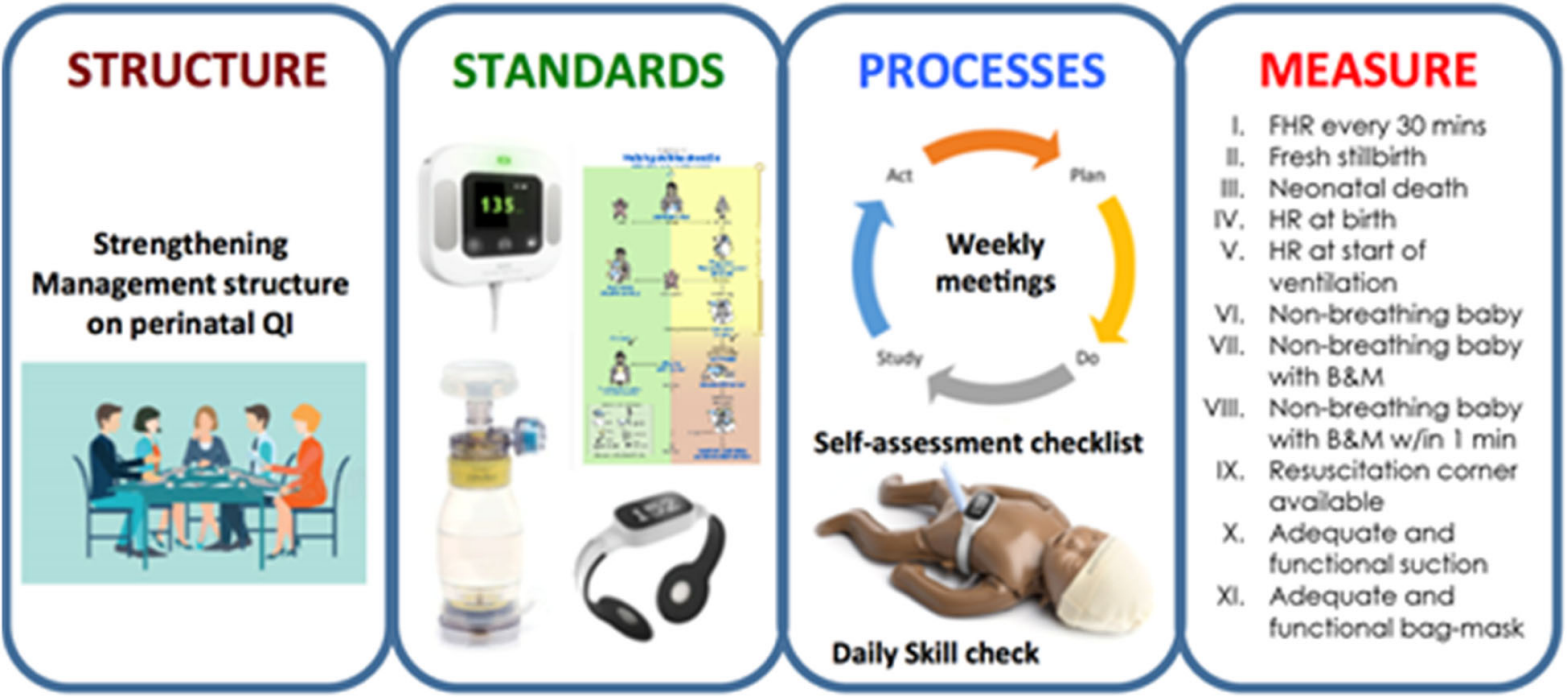
Intervention design of the SUSTAIN package

The implementation research will examine the impact of a new quality improvement project—–the Scaling Up Birth Bundle Through Quality Improvement in Nepal (SUSTAIN). The SUSTAIN project aims to improve intrapartum care through a set of quality improvement interventions (Fig. 1). The interventions are based on learning from our previous implementation research on quality improvement for intrapartum care [29].

The objective of the research is to evaluate:

- The impact of the SUSTAIN package on intrapartum-related mortality;
- The impact of the package on health workers’ performance on monitoring fetal heart rates, essential newborn care, and neonatal resuscitation;
- The appropriateness of implementing the quality improvement interventions in the hospitals [30]; and
- The acceptability of the SUSTAIN package in the hospitals.
- The perception of women for intrapartum care

## Methods

### Trial design

**Fig. 2:**
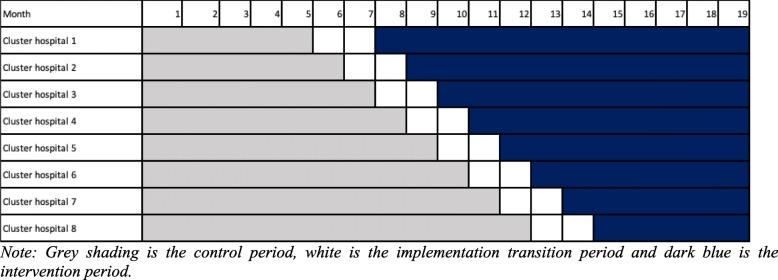
Stepped wedge cluster randomized controlled design. Note: gray shading is the control period, white is the implementation transition period, and dark blue is the intervention period

The trial is a stepped wedge cluster randomized controlled trial in eight public hospitals in Nepal. Each hospital will represent a cluster randomized in a cross-sectional wedge design with intervention transition period in each hospital in 2 months (Fig. 2). With the qualitative research components, we aim to provide insight into women’s and staffs’ response to intervention and to understand how contextual factor affect its implementation [31].

### Settings

Each hospital manages at least 3000 deliveries a year (Table 1). Their intrapartum-related mortality rates range from 11 to 36.5 intrapartum-related deaths (intrapartum stillbirth and first-day mortality) per 1000 births. All the hospitals have separate labor units and operating theaters. Normal and complicated vaginal deliveries take place in the labor units and cesarean sections in the operating theaters. Each hospital has a postnatal unit and a sick newborn care unit. The hospitals provide level II sick newborn care services [32].

**Table 1:** Total birth and mortality rate in the study hospitals (2017)

	Total no. births	Intrapartum stillbirth rate (per 1000 births)	First-day neonatal mortality rate (per 1000 live births)	Intrapartum-related death rate (per 1000 births)
Koshi Zonal Hospital	5464	9.3	4.4	13.7
Janakpur Regional Hospital	14,300	11.9	23	34.9
Bharatpur Hospital	11,006	7.3	3.7	11.0
Lumbini Zonal Hospital	8649	11.2	12.3	23.5
Dadeldhura Sub-Regional Hospital	3112	10.3	4.4	14.7
Mid-Western Regional Hospital	3847	5.0	25	30.0
Bheri Zonal Hospital	4132	1.2	35.3	36.5
Seti Zonal Hospital	6277	2.2	29.7	27.9

Intrapartu-related mortality is the composite of intrapartum stillbirths and first-day mortality data

#### Participants

##### Eligible criteria for participants

The study will cover pregnant women with a gestational age of more than 22 weeks admitted to hospital with fetal heart sound at admission who consent to be enrolled in the study. The process evaluation data are collected from staff members working in the labor and delivery room in the study sites.

#### Interventions

The SUSTAIN package is a bundled QI package to empower health care workers to provide improved care and to review its provision during the intrapartum and immediate postpartum periods. The interventions include training and technology for intrapartum monitoring, neonatal resuscitation, and a supporting system to review the implementation of these measures.

The SUSTAIN interventions in the hospitals will be as follows:

1. The bottleneck analysis of delivery care and setting up mechanisms to review and plan improved care and improve accountability.
2. The introduction of the Safer Births Bundle, which is a set of tools for training and therapy to improve the monitoring of labor (using Moyo FHR monitors®) [33] and neonatal resuscitation (using upright bag-masks® [34], NeoBeat® newborn heart rate meters and NeoNatalie live training manikins®).
3. The implementation of QI interventions in delivery rooms including daily skill checks on neonatal resuscitation, using checklists to prepare for births and resuscitation, using self-review evaluation checklists after neonatal resuscitation incidents, and holding weekly review meetings to track progress on implementing the new tools and standards.
4. The setting up of a system to continuously measure the quality of intrapartum care using the Plan-Do-Study-Act (PDSA) approach [35]. This approach harnesses the local ownership of challenges and provides an actionable framework to monitor and evaluate progress to improve and sustain QI changes.

In the project using the i-PARISH framework, facilitation is done at three different levels of types of facilitators. First, an external facilitator or mentor who introduces the innovation of SUSTAIN to the hospital leadership and conducts an assessment of the hospital context in terms of implementing the innovation. The external facilitator will be an expert to coordinate with the different networks of facilitators. The external facilitators conduct bottleneck analysis workshop, plan meeting, and supervise the internal facilitators. Second, an experienced internal facilitator has a deeper understanding of the organization local context. The facilitator conducts training on the intervention package and the rolls out of the innovations (safer births bundle). Third, an internal new facilitator will be working under the supervision of the experienced internal facilitator to implement the daily skill checks on neonatal resuscitation, using checklists to prepare for births and resuscitation, using self-review evaluation checklists after neonatal resuscitation incidents, and conducting PDSA meeting.

### Outcomes

Primary outcome

- *Intrapartum-related mortality*—intrapartum-related mortality defined as intrapartum stillbirth (no breathing 10 min after delivery) and neonatal death within the first 24 h of life [36].

Secondary outcomes:

- Proportion of deliveries with fetal heart rate monitoring as per standard protocol.
- Proportion of deliveries in which abnormal fetal heart rate during labor is followed by neonatal resuscitation.
- Proportion of deliveries resulting in emergency cesarean sections and instrumental deliveries due to fetal distress.
- Proportion of non-breathing babies who receive a bag and mask ventilation within 1 min of birth.
- Proportion of health workers maintaining neonatal resuscitation skills 6 months after being trained on it.
- Adequacy and acceptability of the implementation of SUSTAIN package

### Sample size

The intervention will be employed in the same hospital. Therefore, every hospital will be included in control as well as intervention groups. This fact will produce the correlated data between the arms. For binary correlated data, we have considered the one-sided McNemar’s test for sample size calculation. Considering the statistical power of 90%, level of significance at 5% and the average cluster size of 4800 deliveries we have calculated the sample size. For adjusting the cluster effect, the sample size is adjusted by the design effect. The design effect is chosen as a proxy of 1.71 based on Nepal’s Demographic Health Survey 2016 [37]. This design effect is updated based on our average cluster size. The design effect for NMR in NDHS 2016 is reported as 1.12 for urban sample with an average cluster size of 799. Based on the information, we have calculated the intra-cluster correlation coefficient (ICC) of 0.00015 which gives the design effect 1.71 for average cluster size 4800 [38]. We have also considered the 7% loss to follow up to reach the final sample size of 31,259 deliveries in each group. This number will be proportionally allocated among the hospitals. The STATA command “power” is used for necessary calculation.

Purposive sampling will be used in the qualitative components of the study. Maximum variation sampling will guide the selection of participants to in-depth interviews with health workers and focus group discussions with the hospital management team. This approach secures a wide variety of people of interest and consequently a broad range of perspectives to better understand contextual factors influencing on implementation. A convenient sample of pregnant women will be interviewed about their experiences of intrapartum care. Sample sizes will be based on data saturation (Table 2).

**Table 2:** Number of health workers in the study hospitals (2017)

	Nurses	Doctors	Other health workers	Total health workers
Koshi Zonal Hospital	25	8	45	78
Janakpur Regional Hospital	60	22	0	82
Bharatpur Hospital	86	20	17	123
Lumbini Zonal Hospital	81	7	36	124
Dadeldhura Sub-Regional Hospital	42	2	13	57
Mid-Western Regional Hospital	20	3	22	45
Bheri Zonal Hospital	33	4	26	63
Seti Zonal Hospital	32	6	56	94

### Randomization

Using a simple random technique, the principal investigator will generate a random sequence among the eight hospitals to determine the temporal stepped wedge sequence of the clusters through simple random technique. The allocation of the introduction of intervention in each hospital will be done in a stepped wedge pattern based on the above randomly generated sequence. Blinding is part of the SW-RCT—clusters/hospitals will not know when the other hospitals will be controls or intervention.

### Data collection

An independent team will be established in each hospital to collect data on implementation, process, and outcomes.

*Implementation level data—*a form will be used to collect data on the number of stakeholders engaged in each intervention:

- A hospital readiness and service availability assessment tool will be used to conduct the bottleneck analysis of service readiness for intrapartum care. A planning tool will be used to develop a plan based on the bottleneck analysis.
- Data on health workers’ knowledge and skills on intrapartum care before and after training will be collected using multiple-choice questions and skills checklist.
- The data on the continuous quality improvement process will be collected using a Plan-Do-Study-Act diary, which was tested in our previous study [21].
- The periodic performance of the health workers will be done using a skills checklist.

*Process level data—*A separate system will be established to collect data on health worker performance in simulated and clinical intrapartum care settings:

- Data will be collected on the use of the NeoNatalie Live® manikin through skill drills in an application-based system.
- The use of fetal heart rate monitor (Neobeat®) will be assessed by a separate group of data collector using an observation checklist
- Data on health worker performance on monitoring fetal heart rates, immediate newborn care, and neonatal resuscitation will be collected using an observation checklist.
- The implementation of the SUSTAIN interventions using the PARIHS framework [39] will be evaluated through in-depth interviews and focus group discussions with service providers and caregivers.
- The acceptability of the new interventions will be assessed by studying health worker acceptance and barriers to use.
- The perceptions of women about intrapartum care will be evaluated with semi-structured interviews.

*Outcome level data—*data on mortality and clinical events during intrapartum care will be collected from patient case notes and labor and delivery registers.

*Socio-demographic data—*the socio-demographic characteristics of the women will be collected through semi-structured interviews.

*Equipment data*—signal data and events that are automatically recorded by the Safer Births equipment will be uploaded to a cloud service with strict access control. Equipment data will be analyzed to complement the process level data and drive local QI processes, they will be analyzed to check the integrity and condition of the equipment and the data will be used to create new insight, develop improved equipment as well new signal analysis methods.

#### Data management

The study will maintain the confidentiality of individual participants including their identity and location. To protect against data loss, all data will be collected in a tablet-based application and kept on a secure server.

In each hospital, a data collection coordinator will assess the quality and completeness of the data. The data collected on a paper-based format from the hospitals will be indexed and a master ID will be provided to each data entry form. Prior to data entry, the completed forms will be reviewed for missing variable(s) and the open-ended responses will be coded. The data will be entered into a CS-Pro database.

#### Data analysis

A data cleaning and data analysis strategy will be developed once the data has been collected. The implementation and process level data will be evaluated using a Medical Research Council process evaluation process [40]. The outcome level data will be analyzed using the CONSORT guidelines for processing quantitative data [41] and COREQ for processing the qualitative data [42].

For the analysis i-PARIHS framework will be used, which essentially involves what is to be implemented, who with, where, and how. The assessment of the evidence of innovation in the SUSTAIN package, characteristics of the different stakeholders to whom the interventions were targeted, characteristics of the settings, and implementation of the facilitation process. The facilitation implemented through a different level of facilitators (external, internal experienced, and new) using different quality improvement interventions to improve the structure, standards, and process will be assessed.

Inductive thematic network analysis will be used to analyze interview data [43]. The audio recorded interviews are transcribed, and verbatim data will be transferred to NVivo Version 12.1.0. The initial coding will be done by research assistants. In the next phase, senior researchers will familiarize themselves with data and critically reviewed codes. Analyses will continue with collating codes in several basic themes, creating organizing themes from basic themes, and developing global theme by combining all data.

#### Ethics

The study protocol has been finalized through a consultative process with professional bodies, academia, global experts, and Nepal’s Ministry of Health and Population. Ethical approval has been received from the Ethical Review Board of the Nepal Health Research Council. Written consent will be taken from all study participants. A data safety monitoring board will be formed to monitor the progress of the research and any deviations from the protocol [44]. The researchers will strive to carry out the study to the highest level of integrity in line with the World Medical Association Declaration of Helsinki Ethical Principles for Medical Research [45].

## Discussion

We will study the impact of an evidence-based package of QI interventions and technologies for improving the quality of intrapartum care and hope that the research will provide useful evidence on a scalable model of QI interventions and technologies for improving intrapartum care. The research will provide new information on what context and intensity of facilitation are required to implement the interventions in hospitals in a low-income setting. The research will evaluate the i-PARISH theoretical framework on the different facilitation strategy through different facilitators to the hospital stakeholders. Furthermore, we aim to construct an evidence-based framework for QI and technology-based solutions to improve intrapartum care. Three tools will be used to translate the evidence into action once the evidence on the process and outcome evaluations are available. First, a plain language summary of the results and on the importance of the QI package will be disseminated to general audiences via the local and national Nepalese media. Second, a brief will be developed for policy-makers outlining the required QI framework for improving care to advocate for more investments in such interventions. Third, the results will be published in a peer-reviewed journal for international academic, researcher, and program experts (Table 3).

**Table 3:** Intensity of each QI item in the SUSTAIN project

Quality improvement package items	Details	Number
Facilitators training on HBS	Training on continuous quality improvement package. Checklist use HBS package	6 days
Continuous quality improvement	An orientation with hospital leadership on the improvement of care process for mothers and newborn	2 h
	Orientation to facilitators on the tools for bottleneck analysis	2–4 h
	Assessment of maternal and newborn services in the hospital	2 days
	Bottleneck analysis of maternal and newborn services	Half day
	Development of problem-solving process using the PDSA approach	2 h
Training	Training health workers on the HBS package and continuous quality improvement (CQI)	3 days
Mentoring	Monthly visit by clinical mentors to guide the implementation of the standards	1 day each month
Audit and feedback	Use of progress board Daily drills	Daily

Contributions to the literature

- Quality improvement packages improve perinatal care in public hospitals.
- New technologies for fetal heart rate monitoring and neonatal heart rate monitoring tend to be well accepted by health care providers.
- To evaluate the adequacy of the implementation of a QI package (the Scaling Up Birth Bundle Through Quality Improvement in Nepal [SUSTAIN] package) in public hospitals using a Stepped Wedge Cluster Randomized Controlled Trial (SW-CRCT).
- The effect of the QI package on health worker’s adherence to intrapartum care (fetal heart rate monitoring and neonatal resuscitation) as per standard protocol and on intrapartum survival.

## Data Availability

Not applicable.

## Abbreviations

CONSORT: Consolidated Standards of Reporting Trials
COREQ: Consolidated Criteria for Reporting Qualitative Research
CS: Pro-Census and Survey Processing System
HBS: Helping babies survive
MDG: Millennium Development Goal
PARIHS: Promoting Action on Research Implementation in Health Services
PDSA: Plan-Do-Study-aAct
QI: quality improvement
SDG: Sustainable Development Goal
SUSTAIN: Scaling Up Birth Bundle Through Quality Improvement in Nepal
